# New Insights on Emotional Contributions to Human Postural Control

**DOI:** 10.3389/fneur.2018.00789

**Published:** 2018-09-21

**Authors:** Allan L. Adkin, Mark G. Carpenter

**Affiliations:** ^1^Department of Kinesiology, Brock University, St. Catharines, ON, Canada; ^2^School of Kinesiology, University of British Columbia, Vancouver, BC, Canada; ^3^Djavad Mowafaghian Centre for Brain Health, University of British Columbia, Vancouver, BC, Canada; ^4^International Collaboration on Repair Discoveries, University of British Columbia, Vancouver, BC, Canada

**Keywords:** postural control, balance, emotions, fear, anxiety, threat, surface height

## Abstract

It has been just over 20 years since the effects of height-induced threat on human postural control were first investigated. Raising the height of the support surface on which individuals stood increased the perceived consequences of instability and generated postural control changes. Since this initial work, converging evidence has accumulated supporting the efficacy of using height-induced threat to study the effects of emotions on postural control and confirming a direct influence of threat-related changes in arousal, anxiety, and fear of falling on all aspects of postural control, including standing, anticipatory, and reactive balance. In general, threat-related postural changes promote a greater physical safety margin while maintaining upright stance. We use the static balance literature to critically examine the current state of knowledge regarding: (1) the extent to which threat-related changes in postural control are sensitive to threat-related changes in emotions; (2) the underlying neurophysiological and cognitive mechanisms that may contribute to explaining the relationship between emotions and postural control; and (3) the generalizability of threat-related changes across different populations and types of threat. These findings have important implications for understanding the neuromechanisms that control healthy balance, and highlight the need to recognize the potential contributions of psychological and physiological factors to balance deficits associated with age or pathology. We conclude with a discussion of the practical significance of this research, its impact on improving diagnosis and treatment of postural control deficits, and potential directions for future research.

## Background

Fear of falling is frequently reported in older adults (1, 2) and patients with balance deficits (3–8) and is a significant predictor of future falls risk (9, 10). Maki et al. (11) were the first to report significant differences in balance control between fearful and non-fearful older adults, followed by evidence of balance control changes in individuals with anxiety disorders and phobias (12, 13). While these observational studies provided important evidence for a link between balance deficits and emotions, such as fear and anxiety, the direction of the relationship was not determined due to limitations of the cross-sectional design (i.e., individuals may be fearful because they have underlying balance deficits, or have balance deficits because they have an underlying fear of falling).

Brown and Frank (14) were the first to use an experimental design to examine the direct effect of postural threat on human balance control. These researchers employed a modified version of an elevated surface height paradigm used extensively to study fear and anxiety behaviors in animals [elevated plus maze, (15)]. When young healthy adults stood on an elevated (0.8 m) platform and responded to an unpredictable forward push to the trunk, they leaned back away from the platform edge and stiffened to constrain the forward movement of the body's center of mass (COM). A series of studies followed to examine the effects of postural threat on standing postural control in young healthy adults (16–18). Collectively, these studies revealed threat-related postural changes that included leaning away from the platform edge (or away from the direction of the perceived threat), and decreased amplitude and increased frequency of center of pressure (COP) displacements during quiet standing. These threat-related responses were more pronounced with the eyes open and when forward stepping was restricted by the edge of the platform (16). Furthermore, these threat-related changes were scaled to the level of postural threat with progressive decreases in sway amplitude and increases in sway frequency observed with increasing surface heights up to 1.6 m (17). The combination of decreased amplitude and increased frequency of COP displacements suggested the adoption of an ankle stiffening strategy (19). With the body modeled as an inverted pendulum when standing quietly, increased muscle activity around the ankle joints would act to tighten control of the COM within the limits of the base-of-support (19, 20). This hypothesis was experimentally confirmed by observations of increased ankle muscle stiffness when standing at height, coupled with EMG changes consistent with increased co-contraction of lower leg muscles, and decreased COM displacements (18). Together, these early studies revealed that threat-related postural changes provided protection against a loss of balance by limiting body position and movement in the direction of the perceived risk associated with the threat. These changes in humans coincide with freezing and stiffening behavior observed in anxious animals when moving on elevated surfaces (21).

Since these initial studies, physically raising the height of the support surface on which individuals stand has been used extensively to: (1) confirm the effects on standing balance control in young and older healthy adults (22–36), and patient populations such as individuals with unilateral vestibular loss (37) and Parkinson's disease (38, 39); (2) extend the effects of threat on different types of postural tasks including anticipatory postural control (34, 40–42), reactive postural control (43, 44), functional balance tasks [e.g., one leg stance; (28)], and normal and adaptive gait (45–53); and (3) explore the neural mechanisms underlying these threat effects (44, 54–67). Studies have also provided converging evidence to confirm that the threat of standing on elevated surfaces (i.e., real or virtual) can evoke psychological (e.g., self-reported increases in perceived anxiety and fear) and physiological responses (e.g., increases in electrodermal activity, blood pressure) typically observed in fearful or anxious conditions [e.g., (25, 28–30, 32, 34, 35, 40)]. Furthermore, significant relationships have been observed between threat-induced emotional as well as cognitive changes (e.g., conscious control of posture) and modifications in postural control [e.g., (28, 30, 64)].

## Evaluating the effects of height-induced postural threat on standing balance control

Given the breadth of research on this topic over the past 20 years, we chose to focus on height-induced postural threat effects on standing balance control, as this represents the majority of studies conducted to date, and has the potential to influence anticipatory and reactive postural adjustments. In order to critically evaluate and allow for a direct comparison between the studies, we controlled for key factors known to influence standing balance control. A search of PubMed, PsychINFO, EMBASE, CINAHL (search terms: postural threat or anxiety, and height, and standing), and hand searches, identified 89 original research articles (non-duplicate). Manual screening removed 51 articles that did not include a manipulation of postural threat/anxiety or a standing task in the study design. The remaining 38 studies were examined, and a subset of studies was selected based on the following five criteria: (1) young or older healthy adults; (2) height threat; (3) quiet standing task; (4) sample duration (≥60 s); and (5) psychological or physiological measure to confirm the efficacy of the threat manipulation. Stance duration was considered a critical factor because it has been shown to significantly affect COP summary measures (68, 69, 70) and varies widely across studies. At least one physiological (e.g., increased electrodermal activity) or psychological (e.g., increased perceived anxiety) measure was required to confirm that the height manipulation generated a significant emotional effect; this was important given the variability of heights and conditions used to manipulate threat across studies. Based on these criteria, eight studies were identified (Table 1), with six studies focusing specifically on young adults (28–30, 32, 34, 35) and two involving older adults (25, 39).

**Table 1:** Height-induced postural threat effects on quiet standing.

**Study**	**Group**	**Maximum threat**	**Sampling duration**	**AP COP MP**	**AP COP MPF**	**AP COP RMS**
Carpenter et al. (25)	14 YA	1.6 m	120 s	Posterior lean	Increased	Decreased
	13 OA	1.6 m	120 s	Posterior lean	Increased	Decreased
Hauck et al. (28)	31 YA	1.4 m	60 s	Posterior lean	Increased	Decreased
Davis et al. (29)	26 YA	3.2 m	60 s	Posterior lean	Increased	Decreased
Huffman et al. (30)	48 YA	3.2 m	60 s	Posterior lean	Increased	*No change*
Pasman et al. (39)	14 OA	1.6 m	120 s	Posterior lean	Increased	*No change*
Cleworth et al. (32)	18 YA	3.2 m	120 s	Posterior lean	Increased	Decreased
Zaback et al. (34)	82 YA	3.2 m	60 s	Posterior lean	Increased	Decreased
Cleworth et al. (35)	20 YA	3.2 m	60 s	Posterior lean	Increased	Decreased

*Table includes studies that met the following criteria: (1) healthy young adults (YA) or older adults (OA), (2) height threat, (3) quiet standing task, (4) sampling duration (≥60 s), and (5) psychological or physiological measure to confirm efficacy of threat manipulation. Significant anterior-posterior (AP) center of pressure (COP) mean position (MP), mean power frequency (MPF), and root mean square (RMS) effects (maximum threat compared to lowest threat condition) for eyes open conditions are reported. Participants stood at the platform edge in the maximum threat condition for all studies except Carpenter et al. (25). Effects reported for Davis et al. (29) do not include results from the fearful sub-group*.

A consistent postural strategy emerged from the collective results of the eight studies that met our criteria. All studies revealed that young and older healthy adults leaned significantly away from the edge of the platform and significantly increased their COP sway frequency (25, 28–30, 32, 34, 35, 39). The majority of the studies also showed that young and healthy older adults decreased their COP sway amplitude (25, 28, 29, 32, 34, 35); two exceptions to this observation reported no significant change in sway amplitude when threatened (30, 39). These observations reinforced the findings of earlier work on standing balance control in young healthy adults (16–18) and extended the findings to older healthy adults. All selected studies were performed with eyes open and gaze fixed on near targets (<4-m) to control for effects of postural height vertigo that may occur with longer (>6-m) viewing distances (71, 72). Yet, similar height-induced postural changes have been observed with eyes closed, and also when peripheral vision was occluded (16, 18, 29). The selected studies focused predominantly on anterior-posterior COP changes (which align with the direction of the threat), with similar effects also reported in the medial-lateral direction, albeit to a lesser degree (17, 31), potentially due to the threat direction (42) or biomechanical constraints of controlling anterior-posterior versus medial-lateral sway (20).

## Context-dependent threat-related changes in standing balance control

Studies have utilized other methods to manipulate threat or emotions, to confirm if the effects of height are generalizable to other threat sources, and to avoid some of the context-specific limitations associated with standing on an elevated surface. One common alternative is to manipulate the threat of an impending perturbation, during which individuals are required to stand with or without the threat of experiencing a sudden, unpredictable balance disturbance, such as a push or pull to the upper trunk (73) or a support surface translation (74, 75) or rotation (57). Like height-induced threat, the threat of perturbation has been shown to significantly increase arousal, anxiety, and fear (57). Using the threat of multi-directional perturbations has the advantage of reducing the likelihood of individuals adopting any directionally specific strategies that are inherent to elevated surface paradigms (14). COP displacements during quiet standing when anticipating the threat of forward or backward perturbation are found to significantly increase in frequency and amplitude, with a significant shift of mean position forward instead of backward (75).

**Figure 1:**
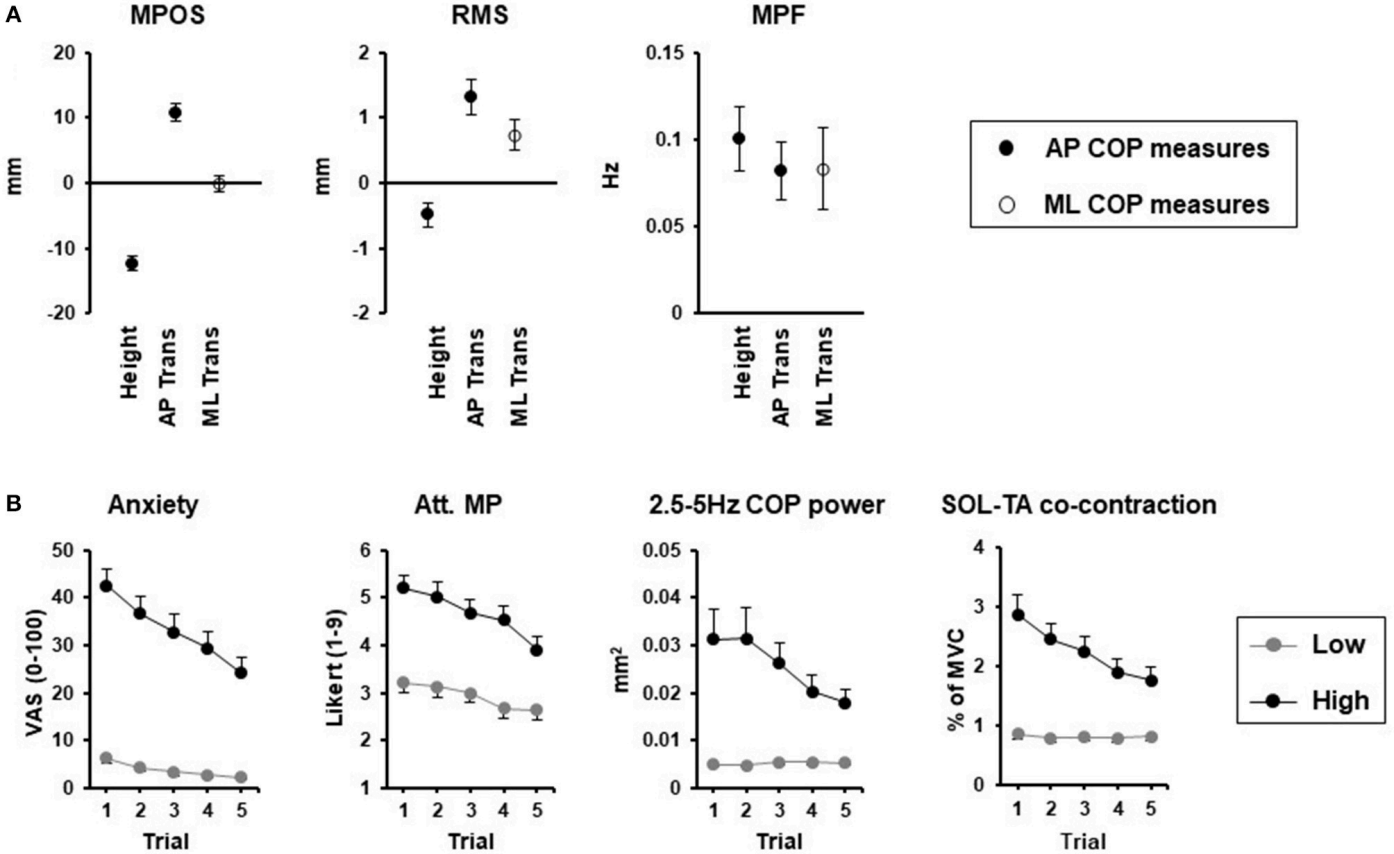
Threat context **(A)** and adaptation **(B)** effects. **(A)** Change in center of pressure (COP) mean position (MP), mean power frequency (MPF), and root mean square (RMS) between threat and no threat conditions for height, [0.8 and 3.2 m surface; 60-s stance duration; modified from (34)] and support surface translations (Trans) in the anterior-posterior (AP) direction [30 s stance duration; modified from (75)] and medial-lateral (ML) direction [60-s stance duration; (76)]. Closed circles reflect AP COP measures while open circles reflect ML COP measures. **(B)** Adaptation of emotional, cognitive (attention focus to movement processes; Att. MP) and postural responses for low (gray circles) and high (black circles) surface heights over 5 repeated 120-s standing trials (77).

Initial comparisons between reported effects of height and perturbation-related threat reveal a common effect of increased frequency of COP displacements during quiet stance (Figure 1A). In contrast, the amplitude of COP displacements and leaning seems context dependent, with smaller amplitude displacements and backwards leaning specific to height-induced threat, and larger amplitude displacements more commonly observed with the threat of a perturbation. While direct comparisons are made difficult by the shorter sample durations typically used in threat of perturbation studies, more recent studies using 60-s durations confirmed the increased amplitude and frequency of COP displacements with this type of threat (76), which are also dependent on the orientation of stance relative to the perceived direction of the threat (Figure 1A). An increase in COP frequency has also been consistently reported in other contexts, including “white coat” effects observed in older women standing under the perceived threat of negative evaluation (78), and young adults standing while viewing affective pictures that elicited increases in arousal, independent of valence (79). In contrast, the increased arousal elicited by mental arithmetic, appears to influence mean position (80), but not COP frequency or amplitude (80, 81), unless coupled with a social evaluative threat (81). Thus, standing balance changes appear to be highly specific to the context, direction, and nature of the perceived threat, which coincides with other threat-avoidance behaviors (82).

## Potential mechanisms underlying threat-related postural changes

The mechanisms that may contribute to, or explain threat-related changes in postural control remain poorly understood. In general, theories can be divided along the lines of emotionally-evoked neurophysiological changes, and/or changes in attention.

Neurophysiological theories are based on the existence of highly-integrated neural networks responsible for processing emotional information, such as fear and anxiety, and sensori-motor control of upright stance (83) and gait (84). Neuro-anatomical evidence for direct influences of emotion onto balance control systems has been well-established in animal models (83, 85, 86). Supporting evidence has been established in standing humans, with threat-induced increases observed in: (1) muscle-spindle sensitivity (56, 57, 87); (2) 1b reflex gain (65); and (3) vestibular gain of balance, head and eye-reflexes (60–63, 66). While early cortical potentials seem unaffected by threat (55, 56), later cortical changes thought to be responsible for processing sensory information are significantly influenced with threat (44, 55, 88).

Alternatively, changes in attention may mediate threat-related postural changes (82, 84). It is possible that threat influences how attention resources are allocated (e.g., individuals choosing to direct attention to their posture) contributing to the postural changes. Huffman et al. (30) demonstrated that with height-induced threat: (1) individuals had a greater tendency to consciously control and monitor their posture; and (2) an increase in conscious control of posture was related to leaning further back away from the platform edge, independent of any changes in amplitude or frequency of COP displacements. Zaback et al. (64) used open-ended questions to categorize how individuals directed their attention under non-threatening and threatening conditions, with five attention focus categories emerging. When standing at a high compared to low height, individuals directed more attention to movement processes, threat-related stimuli, and self-regulatory or coping strategies, and less attention to task objectives and task-irrelevant information. Again, these threat-related attention focus changes were associated with changes in postural control. For example, individuals who directed more attention to movement processes were more likely to demonstrate increases in frequency of COP displacements, and decreases in amplitude of COP displacements when directing less attention to movement processes. In addition, participants that reported increased attention focus to self-regulatory strategies were more likely to show greater decreases in amplitude of postural displacements. Differences in the approach used to assess attention focus in Zaback et al. (64) and Huffman et al. (30) likely contributed to the differences in reported relationships between attention focus and COP measures across studies. This work linking changes in attention focus and postural control provides preliminary evidence that threat-related changes in attention focus may be a mechanism underlying the postural changes (82).

It is most likely that the effects of threat on balance control rely on a complex interaction between neurophysiological changes and changes in attentional processes. With repeated exposure to height, emotional and attentional changes are attenuated, and correspond to reduced changes in high frequency COP displacements and co-contraction of lower leg muscles. In contrast, initial height-induced posterior leaning and decreases in COP amplitude do not appear to attenuate with repeated exposure and thus may be influenced by other mechanisms (e.g., sensory changes, vigilance) not accounted for in the study [Figure 1B; (77)]. Likewise, changes in perception of balance, which relies on a combination of neurophysiological and cognitive-attentional processes, could also contribute to threat-related changes in postural control. Cleworth et al. (35) demonstrated no change in perceived sway (both self-reported, and tracked in real-time using a hand-held device), in contrast to significant reductions in COP and COM amplitude when standing at a high compared to low height. The incongruency of perceived and actual sway with threat (35) mirrors the reported increase in perceived instability of individuals standing on elevated surfaces, despite no change or an actual decrease in sway amplitude (28, 32, 35).

## Clinical relevance

It is crucial to understand how emotional factors can directly and indirectly influence balance control, as these changes have the potential to mask or modify underlying balance deficits. This is particularly important given the high prevalence of fear and anxiety in populations with balance deficits due to age or pathology such as Parkinson's disease, vestibular disorders, stroke, and multiple sclerosis (1–8), and links with postural instability and gait deficits (4, 84). Studies have shown that older adults, individuals with vestibular loss and Parkinson's disease have a similar postural response as young healthy adults to a height-induced threat (24–26, 36, 37, 39). However, the extent to which balance control deficits in these individuals may be attributed to high levels of state and trait fear or anxiety are still unknown (11, 29, 34, 36).

The capacity for fear and anxiety to directly influence balance in healthy adults provides important insight into potential mechanisms through which clinical balance deficits may present without any clear physiological dysfunction. For example, Chronic Dizziness Disorder and Phobic Postural Vertigo (now unified under the diagnosis of Persistent Postural-Perceptual Dizziness; PPPD) are functional dizziness disorders characterized by non-spinning vertigo and subjective balance instability in the absence of any neurological or structural findings, and often have secondary psychological co-morbidities including fear of falling, anxiety or depressive disorders (89). Postural changes in patients with PPPD include increased high frequency (>1 Hz) sway and increased co-contraction of lower-leg muscles under normal standing conditions (90, 91). These changes become less distinct from healthy controls under conditions of threat (92) or attentional distraction tasks (90). These changes correspond to threat-related changes in healthy adults that adapt to repeated exposure and correlate with changes in conscious attention to movement [(77); Figure 1B]. Overall, these observations support the hypothesis that postural changes with PPPD reflect a maladaptation of high-risk postural control strategies triggered by an initial stimulus that persists due, in part to, excessive self-observation and anxiety (89). Likewise, individuals with visual height intolerance (VHI) have been identified in ~30% of the population, defined as those with “an unpleasant feeling caused by visual exposure to heights” (93). When standing on elevated surfaces (15 m), individuals with VHI have increased tibialis anterior activity, greater co-contraction of lower leg muscles, increased ranges of COP sway, and no change in COP RMS (33). However, in the absence of a control group without VHI, it is unclear to what extent the postural changes reported in VHI differ from an otherwise normal manifestation of balance changes observed in healthy individuals standing under conditions of increased postural threat (Table 1).

The potential for fear and anxiety to influence balance is also important to account for when designing intervention studies that require longitudinal measures of balance-related performance in comparison to a baseline measure. Given known white-coat effects (78) and potential first trial effects (17) on balance, it is important that multiple baseline measures be recorded, and/or a control group incorporated to address potential order effects that may be mediated by adaptation of fear/anxiety with repeated exposure (77). Finally, it is important to recognize, and understand, how clinical balance treatments and interventions may be impacted by emotional-balance interactions (83).

## Future research directions

Most studies that used a height manipulation to understand how threat affects normal balance excluded participants with height phobias for safety/ethical concerns; however, there is some reason to believe that a true fear response may have distinct balance changes compared to an anxious response. For example, individuals standing at extreme surface heights [over 9-m high, (22, 23)] are shown to have significant increases in amplitude of postural sway, in contrast to the reduced sway seen in most individuals at lower surface heights (up to 3.2-m high, see Table 1). However, Davis et al. (29) showed that a sub-group of their subjects, who reported a robust fear response to the moderate (3.2 m) surface height threat (>50% change from ground) had a significantly larger amplitude and frequency of COP displacements compared to anxious but less fearful (<50% change) subjects. Thus, more work needs to be done to determine how fear and anxiety may differentially impact unique aspects of balance control, and distinguish those from the context-specific changes which may or may not translate from a lab setting to daily-life situations experienced by those with a fear of falling. Future work also needs to continue to investigate the potential neurophysiological and attentional mechanisms that contribute to postural changes with threat. This includes probing how different sensori-motor systems respond to different threat conditions, and investigating whether specific instructions or tasks designed to shift attention from posture can modify the postural response to threat. Finally, there is a need for exploration of novel techniques such as virtual reality/augmented reality as a means to test and treat individuals with fear of falling, and develop more effective types of balance interventions that are designed to influence both the psychological and physiological aspects of a balance deficit.

## Author contributions

All authors listed have made a substantial, direct and intellectual contribution to the work, and approved it for publication.

### Conflict of interest statement

The authors declare that the research was conducted in the absence of any commercial or financial relationships that could be construed as a potential conflict of interest.
